# Application of Quantitative CT Imaging in Rehabilitation Nursing of Cerebral Apoplexy Patients

**DOI:** 10.12669/pjms.37.6-WIT.4840

**Published:** 2021

**Authors:** Bing Yan, Huanhuan Zhang, Jie Liu

**Affiliations:** 1 Bing Yan, Bachelor’s Degree, Department of Nursing, Huaihe Hospital of Henan University, Kaifeng 475000, Henan Province, China; 2 Huanhuan Zhang, Master of Degree, Department of Nursing, Huaihe Hospital of Henan University, Kaifeng 475000, Henan Province, China; 3 Jie Liu, Master of Degree, Department of Nursing, Huaihe Hospital of Henan University, Kaifeng 475000, Henan Province, China

**Keywords:** Computed tomography, Cerebral apoplexy, Upper limb, Rehabilitation nursing

## Abstract

**Objectives:**

Electronic computed tomography (CT) is an important imaging method for the diagnosis of cerebral infarction. This paper explores the preventive effects of quantitative CT imaging and early rehabilitation nursing on patients with cerebral apoplexy and shoulder-hand syndrome.

**Methods:**

Sixty cerebral apoplexy patients treated were included as control group and given routine care from September 2018 to May 2020. Sixty cerebral apoplexy patients were included as observation group, and early rehabilitation nursing intervention was given based on control group. The incidence of shoulder-hand syndrome and upper limb function were compared between the two groups, to explore the effectiveness of the CT examination in promoting the physical function restoration.

**Results:**

The incidence of shoulder-hand syndrome in observation group after three months of intervention was lower than that in control group, and the severity was less than that in control group (P<0.05); The Ashworth score of muscle tension in observation group after three months of intervention was lower than that in control group, and the simplified FMA score of the upper limbs was higher than that in control group.

**Conclusion:**

Early rehabilitation nursing intervention after CT examination can prevent the occurrence of cerebral apoplexy and shoulder-hand syndrome and improve upper limb function, which is worthy of promotion.

## INTRODUCTION

Cerebral infarction is a common cerebrovascular disease with high morbidity, mortality and disability.^1^ Computerized tomography (CT) is a commonly used auxiliary method for the diagnosis of cerebral infarction, which is accurate and convenient.^2^ Compared with conventional CT, spiral CT can perform volume scanning in a short period of time, reduce the artifacts caused by motion of patients, and significantly improve the spatial resolution and density resolution, so that early detection of diseased patients.^3^,^4^ This review intends to briefly introduce the CT imaging features of cerebral infarction, to explore the effectiveness of the CT examination in promoting the physical function restoration.

Cerebral apoplexy is a common and frequently-occurring disease. It is mainly characterized by ischemic or hemorrhagic loss of brain tissue.^5^ According to reports, the incidence of shoulder-hand syndrome after cerebral apoplexy is 12.5% to 74.1%.^6^ Rehabilitation nursing is an important part of cerebral apoplexy treatment and an effective way to promote the recovery of patients’ neurological and motor functions.^7^ The purpose of this study was to observe the preventive effects of early rehabilitation nursing combined with CT, and to provide a basis for shoulder and hand health education for the prevention and control of cerebral apoplexy.

## QUANTITATIVE CT IMAGE CORRELATION THEOREM

The dataset for the severity scoring (SVR) task is provided by Image CLEF Tuberculosis 2017.^8^ The dataset consists of a total 160 chest CT scans of Tuberculosis patients in addition with clinically relevant metadata. The 3D CT images which were provided have a slice size of 512×512 pixels and a number of slices varying from 50 to 400. All the CT images are stored in NIFTI file format.

### Basic Fuzzy C-means (FCM) Algorithm

The basic Fuzzy C-means (FCM) algorithm scientifically plans the image data set into multiple sub-regions, and then calculates the membership of each key point by means of an iterative algorithm, and constantly revises the cluster center, so that the objective function can achieve the optimal solution.^9^ The specific clustering objective function equation in this way is as follows:


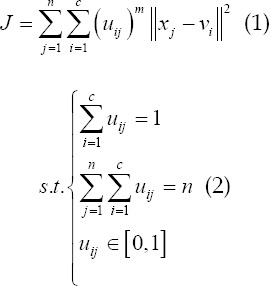


### In the above equation:

fuzzy membership *u_ij_*, which represents the membership of the J data to the I cluster center; *V={v_i_*} is the set of cluster centers; 
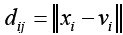
 is the Euclidean distance from the j pixel to the I cluster center the number of clusters c satisfies 1 <c <n; the fuzzy weighting index m represents the fuzzy degree of the fuzzy membership matrix U, and 1≤*m*<∞. The Lagrangian multiplier algorithm is used to obtain the optimal membership and clustering center, which is as follows:


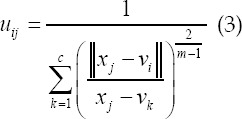



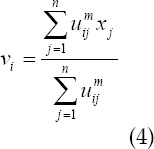


### FCM-S algorithm

The FCM-S algorithm combines neighborhood information in the processing history of each pixel, which effectively compensates the grayscale of uneven images. Its upgraded objective function equation as follows:


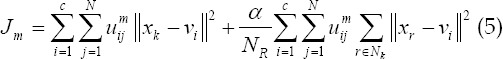


*x_k_* represents the gray value of the k pixel; *v_i_* represents the I cluster center; *U_ik_* represents the membership of the k pixel to the I cluster center; m represents the weighting coefficient of the membership; *N_R_* represents the neighborhood The number of pixels; *N_k_* represents the number of all pixels in the neighborhood of the pixels; *α* represents the penalty factor, which directly determines the degree of influence of the neighborhood on the center pixel.

The above equation is the relationship between the clustering center and the degree of membership:


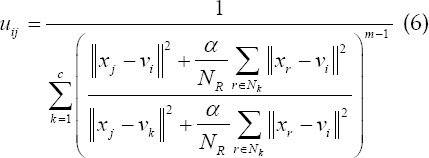



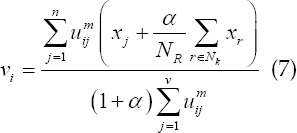


The FCM-S algorithm has good anti-noise performance, but the complexity of the algorithm is very high, which lengthens the calculation time and reduces its overall use efficiency.

## METHODS

Sixty patients with cerebral apoplexy from September 2018 to May 2020 were selected as observation group, with 32 males and 28 females, with an average of (57.42 ± 5.82) years. Sixty cerebral apoplexy patients were selected as control group, 34 males and 26 females, with an average of (58.19 ± 6.23) years.

Cerebral infarction was treated with antiplatelet, nutritional nerve and vasodilator, and cerebral hemorrhage was treated with hemostasis and dehydration. Control group received routine nursing, including correct posture, rehabilitation training, and health education. Based on which, observation group was given early rehabilitation nursing intervention 48 hours after the patient’s condition was stable. Rehabilitation nursing intervention time for both groups was five weeks.

Implement phased rehabilitation interventions based on the patient’s individual upper limb muscle strength.^10^ ***Phase 1:*** Keep the dorsiflexion of the wrist joint as far as possible for 24 hours, and passively move the shoulder strap, elbow joint, and wrist joint of the patient. ***Phase 2:*** Use axillary support when sitting up to prevent subluxation of the shoulder joint; combined with Bo bath technology for handshake assisted exercise training. ***Phase 3:*** Instruct patients to perform turn-up sit-up exercises, healthy and affected side turn-over training, and handshake assisted exercise training combined with Bo bath technology. ***Phase 4:*** Bo bath ball exercises (control key points, posture reflexes), rollers and wooden nails training.

It is treated with an intermediate frequency electric stimulator and the output voltage is 80V. Place a pair of electrodes at 1/3 and 1/2 of the distal end of the forearm. Place another pair of electrodes on the lateral deltoid muscle and supraspinatus. The frequency of the therapy device was adjusted to 4 Hz, and each treatment was performed for 20 minutes twice a day, treated six times a week.

Take one pot of cold water (10°C) and one pot of warm water (60°C), and soak the affected side of the patient’s hand alternately. First immerse the affected hand in warm water for 10 minutes, then cold water for 20 min, 3 times/d. If the patient has severe pain in the affected limb, prednisone can be taken orally, and lidocaine and compound betamethasone can be injected into the joint cavity.

Use a cord (3 to 4 mm in diameter) to wrap from the distal end to the proximal end of the affected limb. Start with the thumb, then the other fingers, and then wrap the palm, back, and wrist in turn. Immediately after the sequential winding, pull the wound cord from the finger loop. 2 times/d, 5d per week.

**Fig.1:**
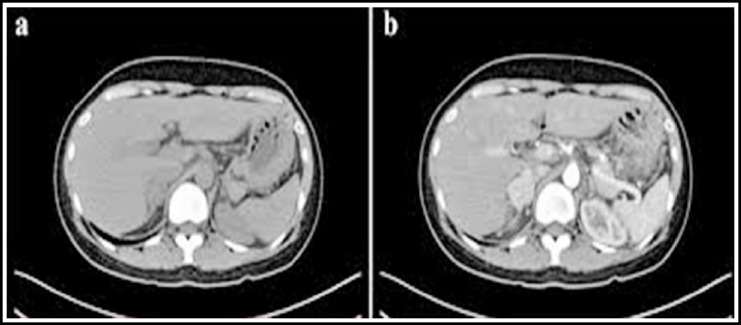
Shadow of dense arteries in brain tissue.

*Low-density stove*. The results of animal experiments show that when the brain tissue is continuously ischemic for more than 8 hours, low-density lesions with sharp borders will appear inside. Local swelling of brain tissue. A CT diagnosis found that patients usually had a placeholder effect within five hours of onset. *Dense arterial shadow*. A small number of patients will have dense arterial shadows within one day of cerebral ischemia with clinical specificity> 90% (Fig.1). Lenticular nucleus sign and insular band sign. These two different signs often appear in the middle cerebral artery, 10 hours after the onset of cerebral infarction.

### Observation indicators

The morbidity and severity of shoulder-hand syndrome at three months after nursing, shoulder mobility and pain before and three months after nursing were measured. According to “Rehabilitation Assessment and Treatment of Cerebral apoplexy”, Grade-I: painful dyskinesia of the shoulder and hand, swelling of the hand; Grade II: muscle atrophy, swelling of the hands, color change, pain dyskinesia; Grade III: obvious muscle atrophy, joint limitation, contracture; Shoulder joint mobility: the normal value is 0 - 180°.^11^ Pain situation: a five-finger pain evaluation model is used, with the little finger as painless, the thumb as unbearable severe pain, the middle finger as moderate pain. The higher the score, the more severe the pain.

The modified spasm scale was used to evaluate the muscle tension of the upper limbs before and 3 months after nursing, the higher the level, the greater the muscle tension. The Fugal-Meyer (FMA) scoring was used to evaluate the upper limb motor function before and three months after nursing.^12^ The higher the score, the more upper limb function it is good.

### Statistical Analysis

SPSS15.0 was used. Measurement data was expressed by (*X̅*±S). Comparison between groups was tested by t. The count data [n (%)] was measured by chi-square test. P <0.05 was considered statistically significant.

## RESULTS

There were no significant differences in shoulder mobility and pain scores between groups before the intervention (P> 0.05); the incidence of shoulder-hand syndrome was lower in observation group after three months of intervention than in control group, and the severity was milder versus control group. The shoulder joint activity was greater than that of control group, and the pain score was lower versus control group, and the difference was statistically significant (P <0.05) (Tables-I and II).

**Table-I:** Comparison of the occurrence of shoulder-hand syndrome [n (%)].

*Group*	*Grade I*	*Grade II*	*Grade III*	*Total*
Control group (n=60)	12(20.00)	4(6.67)	4(6.67)	20(33.33)
Observation group (n=60)	3(5.00)	1(1.67)	0	4(6.67)

**Table-II:** Comparison of shoulder joint mobility and pain scores (*x̅*±*s*).

*Group*	*Shoulder joint mobility / °*		*Pain score / point*	

	*Before nursing*	*Nursing 3 months*	*Before nursing*	*Nursing 3 months*
Control group (n=60)	178.26±1.37	132.59±18.25	0.23±0.06	1.31±0.42
Observation group (n=60)	179.25±2.45	174.21±7.28^*^	0.21±0.07	0.16±0.05^*^

The muscle tension and upper limb function before nursing were not statistically significant (P> 0.05); the Ashworth score of the muscle tension in observation group after 3 months of intervention was lower versus control group, and the simplified FMA score of the upper limbs was higher versus control group. The difference was statistically significant. (P <0.05) (Table-III).

**Table-III:** Comparison of muscle tone and upper limb function (*x̅*±*s*).

*Group*	*Ashworth score*		*Upper limb FMA score*	

	*Before nursing*	*Nursing 3 months*	*Before nursing*	*Nursing 3 months*
Control group (n=60)	1.83±0.82	1.41±0.54	15.72±6.35	18.21±10.53
Observation group (n=60)	1.92±0.75	1.09±0.41^*^	14.56±5.21	23.25±12.41^*^

## DISCUSSION

CT examination results showed that the rate of change of low-density foci of cerebral infarction is relatively fast, and it appears concentrated in the hours after the onset, and the duration is usually> 10d. Within 24 hours after the onset of cerebral infarction, CT images can detect low-density infarcts, but there are some problems of missed diagnosis. Two weeks after the onset of CT, low-density lesions tend to decrease, which is clinically referred to as the “fuzzy effect”. Studies have pointed out that, possible factors are: macrophages continue to extravasate, while capillaries continue to expand, clearing necrotic brain cells; edema at the beginning of cerebral infarction disappears; diffuse hemorrhage at the infarct site makes low-density images gradually disappear.^13^-^16^ There are also studies showing that, multi-mode CT can perform thin-slice, fast, and large-scale scans, greatly shortening the time of CT perfusion imaging, and that it collects the imaging information of acute cerebral infarction lesions more comprehensively, conducive to the clinical selection of appropriate treatment options.^17^,^18^

In the study, compared with conventional care, observation group had better FMA scores, upper muscle tone Ashworth scores, shoulder mobility, and pain scores after intervention, and the incidence of shoulder-hand syndrome was low.^19^,^20^ Precautions will prevent shoulder and hand injuries and compression of the affected limb. The above training can promote the blood and lymph circulation function of the affected limbs of patients with cerebral apoplexy, promote neurological nutrition and metabolism, cause reflex impulse, maintain a certain degree of joint activity, and effectively prevent contracture of muscle ligaments.

## CONCLUSIONS

Microvascular expansion-vasoconstriction response, improve sympathetic tone and relieve muscle tension. The concentric winding method can pressurize the blood flow and the interstitial fluid to return to the heart, suppress the inflammatory exudation of the affected limb, improve blood circulation, reduce pain and edema. CT scan is of great value in the diagnosis of cerebral infarction, and it also plays an important guiding role in promoting the improvement of NIHSS score and the recovery of limb motor function in patients with cerebral infarction. The use of imaging diagnostic technology can provide better guidance for the treatment of patients and effectively assess the prognosis of patients. Early rehabilitation nursing intervention can prevent the occurrence of cerebral apoplexy and shoulder-hand syndrome and improve upper limb function, which is worthy of promotion.

### Authors’ Contribution:

**BY:** Conceived the study, literature review, participated in its design, coordination, analyzed the data and helped to draft the manuscript and also responsible and accountable for the accuracy or integrity of the work.

**HZ:** Helped in design, data collection, article drafting & critical revision.

**JL:** Takes the responsibility and is accountable for all aspects of the work in ensuring that questions related to the accuracy or integrity of any part of the work are appropriately investigated and resolved.
